# Sulfur Analogs of the Core Formose Cycle: A Free Energy Map

**DOI:** 10.3390/life15010001

**Published:** 2024-12-24

**Authors:** Jeremy Kua, Maria T. Peña, Samantha N. Cotter, John Leca

**Affiliations:** Department of Chemistry & Biochemistry, University of San Diego, San Diego, CA 92110, USA

**Keywords:** origins of life, thermodynamics, kinetics, prebiotic chemistry, formose reaction, sulfur

## Abstract

Using computational methods, we examine if the presence of H_2_S can tame the unruly formose reaction by generating a free energy map of the reaction thermodynamics and kinetics of sulfur analogs within the core cycle. With mercaptoaldehyde as the linchpin C_2_ species, and feeding the cycle with CH_2_O, selected aldol additions and enolizations are kinetically more favorable. Thione formation is thermodynamically less favored compared to aldehydes and ketones, but all these species can be connected by enolization reactions. In some sulfur analogs, the retroaldol transformation of a C4 species back into linchpin species is thermodynamically favorable, and we have found one route incorporating where incorporating sulfur selects for a specific pathway over others. However, as CH_2_O diminishes, the aldol addition of larger species is less favorable for the sulfur analogs. Our results also suggest that competing Cannizzaro side reactions are kinetically less favored and thermodynamically disfavored when H_2_S is abundant.

## 1. Introduction

In extant biochemistry, autocatalytic cycles are a key feature of the metabolism [1]. However, outside of living systems, there are few instances of such reaction networks that utilize simple substances not artificially designed. One exception, of interest to the origins-of-life research community, is the formose reaction whereby the C_1_ “food” molecule (CH_2_O) is converted to (CH_2_O)_n_ sugars of increasing size and diversity. In the 1970s, the formose reaction was extensively investigated as a way to boost carbohydrate and food production [2], but ultimately proved unfeasible. The core sugar-forming reaction mechanism utilizing aldol and retro-aldol reactions is now well known [3,4,5]. However, because aldehydes are present, the alkaline conditions used in the formose reaction lead to a complex mixture due to Cannizzaro disproportionation reactions [6], and thus, includes a plethora of acids and polyols.

There has been a recent resurgence in the investigation of the glorious mess that is the formose reaction. In a series of systematic studies, the Huck group examined how the observed product distribution was affected by changing the environmental variables [7,8,9]. Paschek and co-workers examined how catalysts, plausibly present in carbon-containing meterorites, influenced sugar synthesis [10], while Vinogradoff et al. used olivine silicate catalysts [11]. Haas et al. investigated the effects of mechanochemistry [12]. Omran, who highlighted the messiness of the formose reaction [6], more recently looked at the self-construction of chemical gardens under conditions resembling hydrothermal vents [13]. Large-scale computational methods have also been applied to the formose reaction to test various chemical network models [14,15,16].

**Figure 1:**
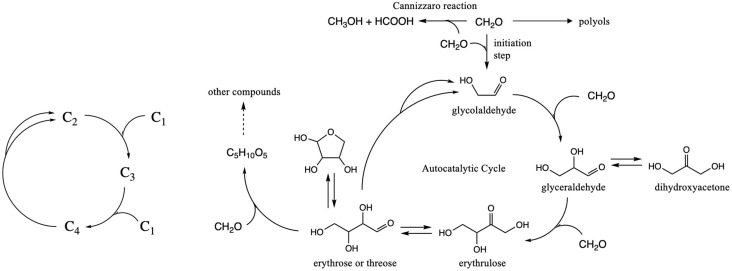
Core autocatalytic cycle of the formose reaction.

Our focus is on the smallest autocatalytic core of the formose reaction, as shown in Figure 1. The C_1_ “food” species is CH_2_O, and in the absence of any other compounds in the autocatalytic cycle, the reaction has a slow induction phase. This is because the direct dimerization of CH_2_O into the C_2_ linchpin molecule glycolaldehyde has a high activation barrier due to the absence of an umpolung species to create new C–C bonds. However, once a small amount of the linchpin is present, the reaction accelerates rapidly. The direct dimerization can now be bypassed because the aldol addition reactions, C_2_ + C_1_ → C_3_ and C_3_ + C_1_ → C_4_, proceed with much lower barriers. Autocatalysis is triggered when a retro-aldol reaction regenerates more of the linchpin species (C_4_ → C_2_ + C_2_) which leads to increasing rates of CH_2_O consumption. While lower concentrations of larger sugars (C_5_ to C_8_) may be observed relatively early in the reaction, most of these are produced in later stages after CH_2_O has been consumed. Note that the Cannizzaro disproportionation reaction is always present, and not just for the C_1_ molecules (as shown in Figure 1); any sugar molecule in the cycle can disproportionate.

In our early work [17] on formaldehyde oligomerization, we found that polyol formation may compete with the aldol addition, but although C–O bond formation is kinetically favored over C–C bond formation, the former is thermodynamically reversible and hydrolyzes in aqueous solution. In contrast, the aldol addition of CH_2_O with C–C bond formation is thermodynamically quite favorable and has significantly higher barriers for the reverse reaction. Also, isomerization reactions (e.g., glyceraldehyde to dihydroxyacetone) or ring closures (e.g., erythrose or threose for the C_4_ species) may lead to an equilibrating pool of off-cycle species that reduce the reactivity of the in-cycle and more reactive aldehyde compounds.

Our group is interested in proto-metabolism. After completing the initial (albeit) small thermodynamic maps of CHO and CHOS compounds [18,19], we wondered if the presence of sulfur could play a role in taming the complex product distribution of the formose reaction. Sulfur has a long, storied history in prebiotic chemistry. While the autocatalytic metabolic core [20] in present life (exemplified by the tricarboxylic acid cycle and its analogs) mainly consists of CHO molecules, the importance of coenzyme A may be a vestige of sulfur’s broader involvement in proto-metabolism as proposed by De Duve [21]. The Sutherland group’s prebiotic map proposes a “cyanosulfidic” world [22], and sulfur’s significance at the origin of life has been highlighted in a recent review [23]. Sulfur was also prominently featured in Wachterhauser’s pyrite world [24], which helped usher proto-metabolism to the forefront of recent origin-of-life research.

As previously reported [19], we identified mercaptoaldehyde as the C_2_ linchpin species analogous to glycolaldehyde, and showed that its formation from glycolaldehyde and H_2_S was thermodynamically favorable and kinetically feasible. We outlined the potential favorable thermodynamics of a subset of the C_3_ and C_4_ sulfur analogs, but we did not examine detailed pathways (the purpose of that paper was to look at broad thermodynamic trends). We speculated that depending on where the thiol groups were positioned, they may provide some degree of selectivity but we only provided broad trends: thiol groups on terminal carbons were thermodynamically favored, and sulfur in the aldose rings could shift the equilibrium from exclusively favoring ketoses over aldoses. We did not look at sulfur’s influence on the retroaldol reaction, nor calculate most of the reaction barriers that would influence the kinetics of the aldol additions, isomerizations, or Cannizzaro reactions.

The present work takes a detailed look at the core autocatalytic cycle and examines how sulfur analogs influence the thermodynamics and kinetics of the many interconnected reaction steps. Thus, we provide an analogous (albeit messier) map to our recent study of the smallest core [25]. While H_2_S was mentioned in the previous work, it was only in the context of sequestering formaldehyde or as an external catalyst rather than being incorporated as thiols throughout the cycle.

This article is organized as follows: After describing our computational protocol and its limitations, the combined results and discussion addresses (1) how the presence of H_2_S impacts the C_1_ food and C_2_ linchpin compounds; (2) the competing Cannizzaro reactions drive the reduction of food species to methanol; (3) the impact of thiols in the two aldol reactions that add CH_2_O to form C_3_ and C_4_ species, respectively; (4) how thiols may shift the favorability of the C_4_ → C_2_ + C_2_ retroaldol; and (5) we will examine the potential of C_5_ and C_6_ forming reactions and their limitations.

## 2. Computational Methods

We use the same computational protocol as our recent work on the thermodynamic map of small CHOS molecules and the exploration of the core formose cycle [19,25] so we can make direct comparisons and extend our free energy map. Here, we provide a brief description of that protocol for convenience. Much of the text in this section is reproduced from those two articles (published in this journal) [19,25] since we think the description is both clear and succinct. Essentially, we calculate the free energies using quantum chemical methods, and our protocol showed good agreement with the available experimental results for CHO systems [17,18,26,27] (there are no experimental data for sulfur-containing compounds involved in the formose reaction).

The computational details are as follows: The geometry of each molecule is optimized and its electronic energy is calculated at the B3LYP [28,29,30,31] flavor of density functional theory with the 6-311G** basis set. To maximize the probability of finding the global minima, multiple conformers are generated using molecular mechanics (MMFFs force field [32]). The optimized structures are embedded in a Poisson–Boltzmann continuum to calculate the aqueous solvation contribution to the free energy. While this does not provide a specific concentration, it assumes a dilute solution such that the electrostatic field generated by a neighboring solute molecule is effectively screened by the water solvent. One can consider all solutes to have the same relative concentrations in our calculations. In a handful of cases, when the solvation calculation gave a seemingly spurious free energy, we made empirical corrections as explained in the Supplementary Materials.

Zero-point energy corrections are included, and we apply the standard temperature-dependent enthalpy correction term (for 298.15 K) from statistical mechanics by assuming translational and rotational corrections are a constant times *kT*, and that low frequency vibrational modes generally cancel out when calculating enthalpy differences. However, entropic corrections in aqueous solution are problematic [33,34,35]. Changes in free energy terms for translation and rotation are poorly defined in solution due to restricted complex motion, particularly as the size of the molecule increases (thus, increasing its conformational entropy). Free energy corrections come from two different sources: thermal corrections and implicit solvent. Neither of these parameters is easily separable, nor do they constitute all the required parts of the free energy. We follow the approach of Deubel and Lau [36], assigning the solvation entropy of each species as half its gas-phase entropy (calculated using standard statistical mechanics approximations similar to the enthalpy calculations described above), based on proposals by Wertz [37] and Abraham [38] that upon dissolving in water, molecules lose a constant fraction (~0.5) of their entropy.

To estimate activation energies, transition states were optimized by including several explicit water and/or catalytic molecules to aid in transferring H moieties. All calculated transition states have one significant negative eigenvalue corresponding to the reaction coordinate (eigenvector) involving bond breaking/forming. Several conformers built by hand are tested in each case and we only report the lowest calculated barriers.

In comparing the equilibrium concentrations in a self-oligomerizing solution of 1 M glycolaldehyde at 298 K, our protocol fared very well compared to subsequent NMR measurements [27]. Our relative Gibbs free energies in aqueous solution are typically within 0.5 kcal/mol compared to experiment. That being said, our protocol shows systematic errors of 2–3 kcal/mol when calculating barriers involving carbonyl chemistry when compared to experimental results. Going to a higher level of theory does not reduce this error [39], nor does using anionic species [18]. There are also specific computational problems that include cations in our protocol, as discussed in previous work [25]. Quantum chemistry is about error cancelation, and our protocol (with its foibles, including the simplistic entropy correction) has worked well even with this systematic error for activation barriers. Thus, we do well on thermodynamics, while we have a larger error bar for kinetics though still reasonable for the carbonyl chemistry and aldol reactions in this work.

## 3. Results and Discussion

To connect this work to our previously published CHOS thermodynamic map [19], we will use the same set of reference compounds: CO_2_, H_2_, H_2_O, and H_2_S will be assigned a relative free energy, *G*_rel_ of 0.0 kcal/mol. The *G*_rel_ of all other species can be determined by calculating the change in free energy, Δ*G*, for forming the species, analogous to a free energy of formation. For example, the formation of the linchpin species mercaptoaldehyde (C_2_H_4_OS) can be written as follows:

2 CO_2_ + 4 H_2_ + H_2_S → C_2_H_4_OS + 3 H_2_O

Since Δ*G* of this reaction is −6.2 kcal/mol, we assign *G*_rel_(C_2_H_4_OS) = −6.2 kcal. For the rest of this paper, we will use the unit kcal as shorthand to signify kcal/mol.

A consistent set of reference compounds allows us to globally compare energies. In our figures, *G*_rel_ values are found next to each compound and in square brackets next to an arrow for transition states. Since some reactions may have more than one non-reference compound, we will also use Δ*G* to designate the change in free energy when focusing on a particular reaction, where Δ*G* = *G*_rel_(products) − *G*_rel_(reactants). Similarly, when we refer to the barrier of a specific reaction, we will designate this Δ*G*^‡^ which compares *G*_rel_ of the transition state to either the reactants or products depending on whether the forward or reverse reaction is being discussed. Throughout this section, we will regularly compare our Δ*G* and Δ*G*^‡^ values to their non-sulfur counterparts in our previous work [25].

### 3.1. Formaldehyde: The “Food” Species

**Figure 2:**
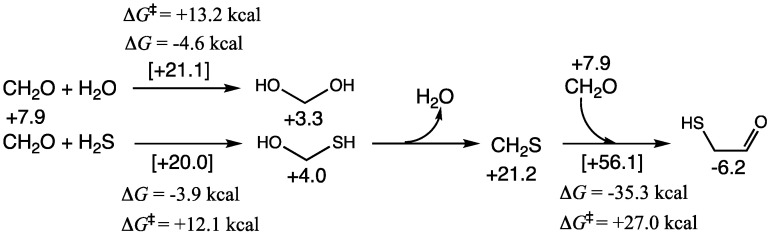
Reactions of CH_2_O in the presence of H_2_S.

As shown in Figure 2, *G*_rel_ of CH_2_O is +7.9 kcal. As shown in our previous work [25], it is thermodynamically favorable for CH_2_O to exist predominantly as its hydrate in aqueous solution. CH_2_(OH)_2_ has a *G*_rel_ of +3.3; therefore, Δ*G* for hydration is −4.6 kcal; the transition state has a *G*_rel_ of +21.1, the barrier Δ*G*^‡^ is +13.2 kcal; thus both our Δ*G* and Δ*G*^‡^ values are in good agreement with experimental values [40].

If H_2_S is present, it can potentially compete with water and add to the carbonyl. Addition of H_2_S is marginally less exergonic (Δ*G* = −3.9 kcal) with a marginally lower barrier (Δ*G*^‡^ = +12.1 kcal). Thus, depending on the concentration of H_2_S we expect to see both addition products in solution in equilibrium with CH_2_O. The dehydration of CH_2_(OH)(SH) to form the thione CH_2_S is significantly uphill (Δ*G* = +17.2 kcal); any CH_2_S formed would easily rehydrate. However, under dehydrating conditions, or if CH_2_S is ever found in higher than transient concentrations, its direct reaction with CH_2_O to form mercaptoaldehyde is highly exergonic, although the barrier is high; *G*_rel_ of the transition state is +56.1 kcal and this reaction is kinetically rather unfavorable.

**Figure 3:**
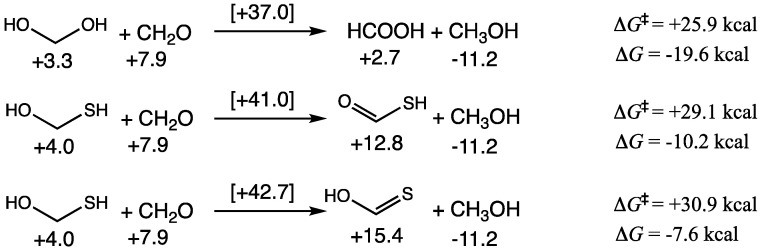
C_1_ Cannizzaro reactions in the presence of H_2_S.

CH_2_O and its hydrate can undergo a Cannizzaro disproportionation reaction to form HCOOH and CH_3_OH. This reaction is thermodynamically favorable (Δ*G* = −19.6 kcal) but has a modest barrier (Δ*G*^‡^ = +25.9 kcal). Thermodynamic favorability is essentially driven by the reduction of CH_2_O to CH_3_OH with a *G*_rel_ difference of −11.2 − (+7.9) = −19.1 kcal, while the oxidation of methanediol to HCOOH has a tiny *G*_rel_ difference. With H_2_S, we expect some concentration of CH_2_(OH)(SH) to be present, thus, the Cannizzaro reaction can lead to a thioacid or a thione-acid as shown in Figure 3. Both reactions are exergonic (although significantly less so) and the barriers are ~5 kcal/mol higher. This is consistent with our thermodynamic map on CHOS compounds where both thioacids and thioneacids were significantly less stable than their corresponding carboxylic acid [19].

**Figure 4:**
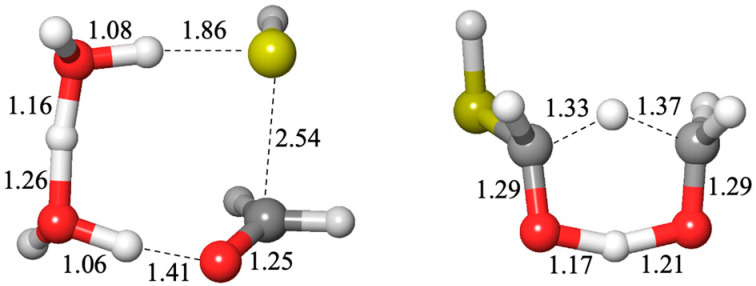
Transition states for adding H_2_S to CH_2_O and a Cannizzaro reaction (bond distances in Å).

The sulfur analogs for addition to carbonyl or a Cannizzaro reaction have similar transition states to their non-sulfur analogs, the hydration reaction or the carboxylic-acid Cannizzaro-forming reaction. The optimum transition state for an addition reaction has two catalytic water molecules (an 8-center transition state) while the lowest barrier Cannizzaro has zero catalytic waters (a 6-center transition state) as shown in Figure 4.

### 3.2. Mercaptoaldehyde: The C_2_ Linchpin Species

**Figure 5:**
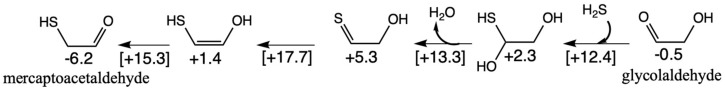
Formation of mercaptoaldehyde from glycolaldehyde.

Glycolaldehyde is the linchpin C_2_ species in the formose reaction; only a small amount is needed to kick-start the autocatalytic cycle (the presence of any member of the cycle will also suffice). For the non-sulfur analog, we have examined its role using the same protocol in our previous work [25]. In the presence of H_2_S, glycolaldehyde can be converted into its sulfur analog, mercaptoaldehyde. The energetics of the reaction pathway was shown in our previous work [19] and is repeated in Figure 5. The reaction is overall exergonic by 5.7 kcal. The first two steps, addition of H_2_S followed by dehydration are slightly endergonic but the barriers are low. The subsequent two steps, conversion of thione to enol to aldehyde, are both exergonic and also have low barriers. Thus, this reaction is both thermodynamically and kinetically feasible.

**Figure 6:**
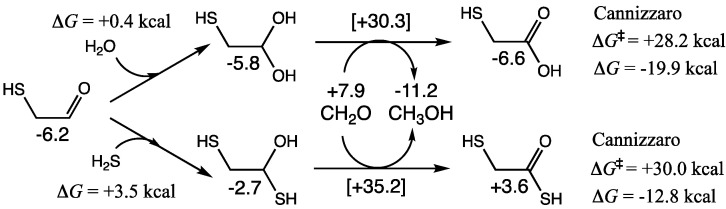
Addition reactions and Cannizzaro reactions of mercaptoaldehyde.

As shown in Figure 6, hydration of mercaptoaldehyde is marginally endergonic by 0.4 kcal. In a dilute aqueous solution, the equilibrium will shift towards the hydrated species. In the presence of food species, the hydrate can undergo a cross-Cannizzaro reaction with CH_2_O to form mercaptoacetic acid. Δ*G* of this reaction is −19.9 kcal (similar to the C_1_ Cannizzaro), while the barrier (Δ*G*^‡^ = +28.2 kcal) is higher by ~2 kcal. Mercaptoaldehyde can undergo a self-Cannizzaro reaction or a cross-Cannizzaro with glycolaldehyde, but both these have higher barriers.

Addition of H_2_S to mercaptoaldehyde is uphill (Δ*G* = +3.5 kcal), and the subsequent cross-Cannizzaro reaction with CH_2_O to form 2-mercaptothioacetic acid is not as exergonic (Δ*G* = −12.8 kcal) and has a higher barrier (Δ*G*^‡^ = +30.0 kcal); C_2_ species containing two sulfur atoms are minor at best (or more likely not found) in the complex mixture.

The way forward into the autocatalytic cycle is the aldol addition of CH_2_O to mercaptoaldehyde via its enol. The C_2_ enolization is 7.6 kcal uphill and the barrier is +21.5 kcal (in Figure 5 on the left, starting from mercaptoaldehyde, this is the reverse step). This is similar thermodynamically to the enolization of glycolaldehyde (Δ*G* = +7.6 kcal, Δ*G*^‡^ = +24.3 kcal) but kinetically mercaptoaldehyde enolization has a lower barrier of ~3 kcal. Hence, the presence of sulfur analogs may accelerate entry into the autocatalytic cycle.

### 3.3. Sulfur Analogs of the C_3_ Species: Formation and Interconversion

**Figure 7:**
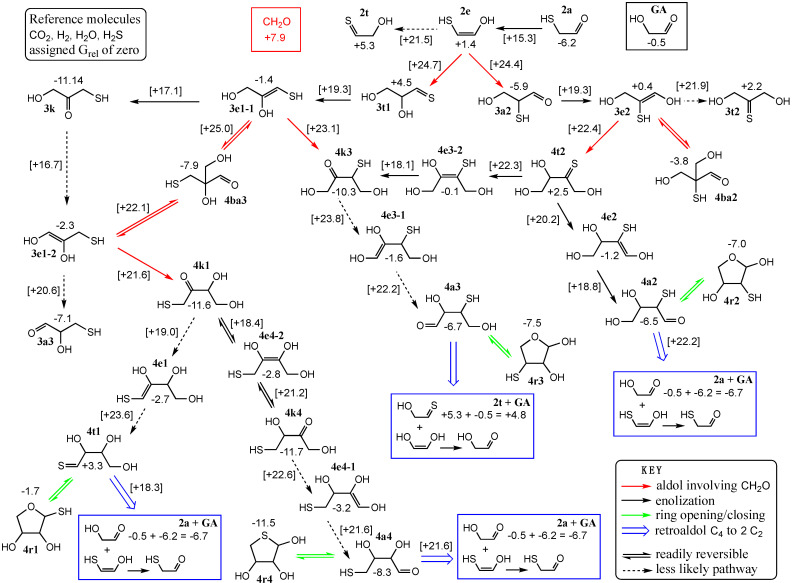
Overall relative free energy map of sulfur analogs and their possible reactions. Blue boxes show retroaldol products.

Before launching into the details, Figure 7 shows our big-picture map of the many reactions that can take place involving C_1_ to C_4_ species that could be involved directly or indirectly in the core autocatalytic cycle. The top row shows the relevant C_1_ and C_2_ species. The second row and the top half of the leftmost column are the C_3_ species. The rest of Figure 7 contains the C_4_ species with retro-aldol products shown in blue boxes. All numerical values (in kcal) are *G*_rel_ of the species (if next to a structure) or a transition state (if next to an arrow and in square brackets). The nomenclature of each compound is based on how many carbon atoms it has, its main functional group, the location of the sulfur, and in some enols, the location of the double bond. For example, the C_2_ species aldehyde, enol, and thione are named **2a**, **2e**, and **2t** respectively. Black arrows refer to enolization reactions. Aldol additions are shown with red arrows. The aldol addition of CH_2_O to **2e** has two possible products, the thione with sulfur on the first carbon (**3t1**) and the aldehyde with sulfur on the second carbon (**3a2**). Further nomenclature will be discussed as we cover the relevant compounds and reactions.

The two possible products for this first C_2_ + C_1_ → C_3_ aldol addition are 2-thioglyceraldehyde (**3a2**) and the thione analog of glyceraldehyde (**3t1**). Stereochemically, these are the analogs of D-glyceraldehyde in conjunction with our previous work [25]. Forming the aldehyde is thermodynamically very favorable (Δ*G* = −5.9 − (1.4 + 7.9) = −15.2 kcal) from the enol (or (Δ*G* = −5.9 − (–6.2 + 7.9) = −7.6 kcal from the aldehyde). This is 2.4 kcal less exergonic than its non-sulfur counterpart, the addition of CH_2_O to glycolaldehyde. However, the barrier for the sulfur analog (Δ*G*^‡^ = +16.5 kcal from the enol) is ~3 kcal lower than its non-sulfur counterpart. Thus, not only is forming the C_2_ enol enhanced kinetically by the presence of the thiol group, but the subsequent aldol addition is also enhanced kinetically. Note that we use the enol rather than an enolate in aldol reactions because our calculations with neutral molecules gave far better results than using anions (see Computational Methods), similar to our previous calculations on the formose reaction [25].

**Figure 8:**
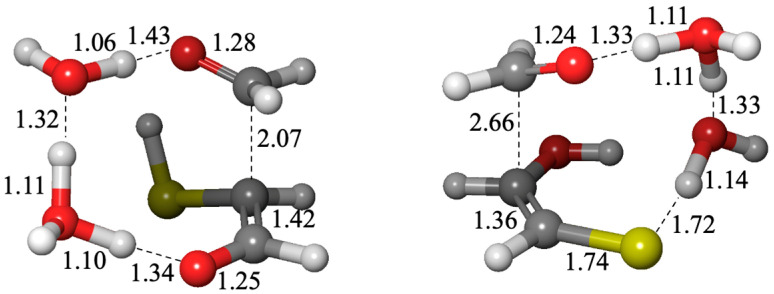
Transition state structures for the C_1_ + C_2_ → C_3_ aldol addition. (Left: **2e** + CH_2_O → **3a2**, Right: **2e** + CH_2_O → **3t1**).

Not surprisingly the thione product (**3t1**) is less favored thermodynamically, but the barrier to form the thione (Δ*G*^‡^ = +16.8 kcal from the enol) is essentially similar to forming the aldehyde. Thus, we expect both C_3_ products to be formed in this system. Interestingly, the transition states have very different distances for the forming C–C bond as shown in Figure 8. In both cases the H transfer is essentially completed before the C–C bond is formed; however, the formation of **3a2** has a shorter distance of 2.07 Å in the transition state, while the less concerted **3t1** has a forming C…C distance of 2.66 Å. We tried several transition state conformations; the structures shown in Figure 8 are the ones with the lowest barriers. Note that the *G*_rel_ values for these transition states at +24.3 and +24.7 kcal are some of the most positive, and therefore, in the overall map, this C_2_ + C_1_ → C_3_ aldol addition may represent the rate-determining step globally.

Interconversion of the C_3_ species to their isomeric counterparts is possible via enolizations. **3a2** can enolize into **3e2** (Δ*G* = +6.3 kcal, Δ*G*^‡^ = +25.2 kcal) but is much less likely to form the thione **3t2** (as indicated by the dashed arrow) which is less favored both kinetically and thermodynamically. The enol is more likely to revert back to the aldehyde **3a2**. On the other hand, **3t1** favorably enolizes to form **3e1-1** (C_3_ enol with thiol on the first carbon, and the double bond at the first carbon). The reaction is 5.6 kcal downhill and the barrier is low (Δ*G*^‡^ = +14.8 kcal) due to the instability of the thione which can be considered a higher-energy or “activated” species. The enol favorably converts to the ketone **3k**, the thermodynamic sink of the C_3_ species. While the ketone could enolize at the other end to form **3e1-2** and subsequently **3a3** (3-mercaptoglyceraldehyde), this is overall less favorable. Thus, **3a3** may be a minor species in equilibrium with **3k**.

**Figure 9:**
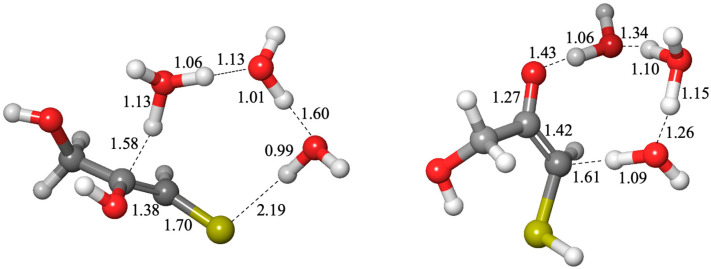
Examples of cis (**3t1** → **3e1-1**) and trans (**3e1-1** → **3k**) enolization transition states.

While we calculated both the cis and trans enols and their corresponding transition states, we found that in the vast majority of cases, the cis enol was favored both kinetically and thermodynamically; hence, we show only the cis isomers in Figure 7 with their corresponding *G*_rel_ values. The free energy differences comparing cis and trans structures can be found in Supplementary Materials. In Figure 9, we show an example of a cis transition state (interconverting **3k** and **3e1-1**) and a trans transition state (interconverting **3t1** and **3e1-1**). The C…H distance in both transition states is similar (1.58 and 1.61 Å). Most of the O…H distances are in the expected range, except the transition state on the left has one that is noticeably longer (1.60 Å) and one noticeably shorter (0.99 Å), and this is likely due to the longer S…H distance of 2.19 Å.

Globally in our map, the *G*_rel_ values for the enolization transition states range from +16.7 to +21.9 kcal. Thus, if the C_1_ + C_2_ → C_3_ barrier can be traversed under some experimental conditions, we expect these enolization reactions to also be kinetically accessible. If the C_3_ enols are formed transiently, and the food species CH_2_O is plentiful, C_1_ + C_3_ → C_4_ aldol addition will proceed. Globally, these aldol addition transition states have *G*_rel_ values ranging from +20.4 to +25.0 kcal, which are slightly higher than for the enolizations. It is more kinetically favorable for a C_3_ enol to convert back to a ketone or aldehyde, but the aldol addition is more thermodynamically favorable, as discussed in the next section.

### 3.4. Sulfur Analogs of the C_4_ Species: Formation and Interconversion

Each of the three C_3_ enols can potentially undergo the aldol addition with CH_2_O to form a C_4_ compound. On the right hand side of Figure 7, **3e2** can either form the branched aldehyde **4ba2** or the thione **4t2**. While the formation of **4ba2** is exergonic, it is a “dead end” where the formose reaction is concerned, and its only route back into the cycle is the reverse retroaldol back to C_3_ and C_1_. Forming **4t2** is only slightly exergonic from the enol (Δ*G* = +2.5 − (0.4 + 7.9) = −5.8 kcal) and barely endergonic from the aldehyde **3a2** (Δ*G* = +0.5 kcal). Not surprisingly, the non-sulfur counterpart forming the ketone is significantly more exergonic. The **3e2** + CH_2_O → **4t2** addition has a relatively low barrier (Δ*G*^‡^ = +22.4 − (0.4 + 7.9) = +14.1 kcal from the enol, or +20.4 from the aldehyde **3a2**). We were unsuccessful in isolating the transition state to form **4ba2**, and our optimizations went to the transition state for the formation of **4t2**. In the non-sulfur counterpart, the barrier to the ketone is 4 kcal lower than to the branched aldehyde. The presence of the sulfur loosens the transition state and is likely why we were unable to isolate the transition state to **4ba2**. Regardless, we do not expect **4ba2** to play an important role in this system.

On the left hand side, CH_2_O addition to the enol **3e1-1** leads to the branched aldehyde **4ba3** or the ketone 3-thioerythrulose (**4k3**). Forming the ketone is both thermodynamically and kinetically more favorable (Δ*G* = −16.7 kcal, Δ*G*^‡^ = +16.6 kcal) from the enol. Forming the branched aldehyde is ~2 kcal less exergonic and the barrier is ~2 kcal higher. However, globally, aldol addition is kinetically less favored than enolization when comparing the transition state *G*_rel_ values as discussed earlier. Similarly to what we see for **3e1-1**, the enol **3e1-2** can add CH_2_O to form the same branched aldehyde **4ba3** or 1-thioerythrulose (**4k1**). Once again, forming the ketone is both thermodynamically and kinetically more favored (Δ*G* = −17.2 kcal, Δ*G*^‡^ = +16.0 kcal) from the enol. Should the branched aldehyde **4ba3** form, it can undergo a retroaldol, eliminating CH_2_O to form either enol, although the path to **3e1-2** is kinetically slightly favored over **3e1-1**.

Similarly to what we found for the C_3_ species, the ketones are the thermodynamic sink for the C_4_ compounds. **4k1** is unlikely to isomerize into the much less stable thione **4t1**, and it most likely equilibrates with **4k4** in solution (with a computationally insignificant *G*_rel_ difference of 0.1 kcal). **4k4** can isomerize into the aldehyde **4a4** (via a terminal enol) and a small amount of the aldehyde likely exists at equilibrium. Similarly, the ketone **4k3** can isomerize into the aldehyde **4a3** (center of Figure 7) as a minor species at equilibrium. On the right hand side of Figure 7, the less stable thione **4t2** has two pathways forward. It could isomerize to the ketone **4k3** or to the aldehyde **4a2**. Both pathways are rather exergonic, with the ketone being thermodynamically favored over the aldehyde. However, the path to the aldehyde (via enol **4e2**) is favored kinetically.

While we expect the open chain aldehydes **4a3** and **4a4** to be minor species in solution equilibrating with their respective ketones **4k3** and **4k4**, once **4a2** is formed it is unlikely to reverse to the much less stable thione **4t2**. This pathway will be of particular interest in light of the C_4_ → 2 C_2_ retro-aldol that facilitates autocatalysis to be discussed in the next section. Also, any of the open chain aldehydes can be partially sequestered by ring-closing reactions to form the furanoses (**4r2**, **4r3, 4r4**). The furanoses are all slightly favored thermodynamically over their open chain aldehyde counterparts and the barriers to ring closure (and ring opening) are low (~12–16 kcal) and similar to aldehyde hydration barriers [17]. We expect these furanoses to be part of an equilibrating mixture. Since the formation of the aldehyde-thione **4t1** is less likely, we do not expect to see much of its ring-closed counterpart **4r1** either.

All values and structures shown in Figure 7 are sulfur analogs for D-erythrose and D-erythrulose. While we also calculated the *G*_rel_ values for D-threose, the overall story does not change and the differences in energies only show minor variances of ~0–2 kcal/mol. Thus, we do not include the threose/erythrose differences in the main body of this article to keep the discussion tractable. The relevant data for D-threose sulfur analogs can be found in Supplementary Materials.

### 3.5. Tetrose Aldol and Retro-Aldol Reactions

In the non-sulfur formose reaction, the (D-erythrose) C_4_ → 2 C_2_ retro-aldol reaction is endergonic by 2.2 kcal. Hence, autocatalysis likely does not kick in until there is a sufficient concentration buildup of C_4_ versus C_2_. The barrier is high at 31.5 kcal. Can the presence of sulfur change this situation?

Of the four retroaldol reactions in Figure 7 (blue arrows and boxes), only one is significantly exergonic: **4t1** → **2a** + **GA** (glycolaldehyde). The split initially produces **GA** and the enol of mercaptoaldehyde that easily enolizes into **2a**. Δ*G* = −10.0 kcal for the overall reaction and the barrier is low (Δ*G*^‡^ = +15.0 kcal) because the reactant is an activated species. However, it is very unlikely that high-energy **4t1** is formed in the first place, and we do not expect this pathway to be practically realized (For all our C_4_ → 2 C_2_ retro-aldol reactions, the barriers to erythrose were consistently lower than for threose by 2–3 kcal).

We expect some (albeit small) amount of **4a4** to be present in the mixture since it can be formed from the ketone thermodynamic sink **4k4**. The reaction **4a4** → **2a** + **GA** is endergonic by 1.6 kcal/mol, marginally less unfavorable than its non-sulfur counterpart, and the barrier is also marginally lower (Δ*G*^‡^ = +29.9 kcal). Perhaps sulfur can accelerate this autocatalytic step ever so slightly. Unfortunately, the same cannot be said for **4a3** (formed in equilibrium with **4k3**) because the retroaldol split leads to the thione **2t**, and thus, the reaction is significantly endergonic (Δ*G* = +11.5 kcal). We were unable to cleanly locate the transition state because it forms a four-membered heterocycle intermediate that looks like the cycloaddition product of **2t** and the enol of glycolaldehyde. More details are shown in Supplementary Materials, but this pathway is unlikely to occur.

**Figure 10:**
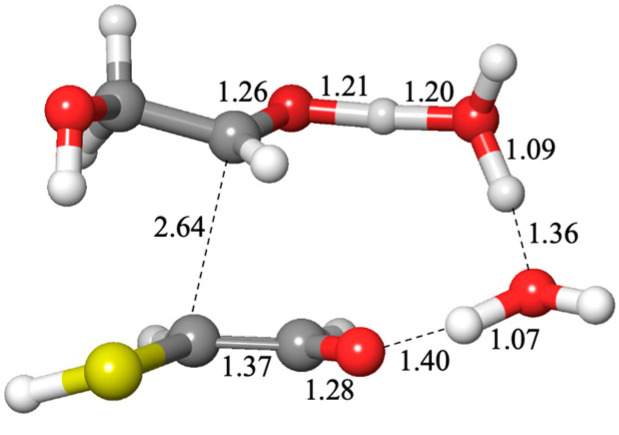
Transition state for the slightly exergonic retroaldol C_4_ → 2 C_2_ reaction (**4a2** → **2a** + **GA**).

The most interesting retroaldol reaction is **4a2** → **2a** + **GA**. From our calculations, the reaction is marginally exergonic (Δ*G* = −0.2 kcal) although this is within the computational error so we consider it equal. The barrier is still relatively high (Δ*G*^‡^ = +28.7 kcal) although lower than the **4a3** retroaldol, and it is ~3 kcal lower than its non-sulfur counterpart. Thus, the situation is more promising in the presence of sulfur when the linchpin mercaptoaldehyde is present. Autocatalysis could begin earlier because the C_4_ → 2 C_2_ reaction is no longer endergonic, and the kinetics are slightly more favorable. The transition state for this retroaldol reaction is shown in Figure 10. It has a longer C…C distance of 2.64 Å and the H has not quite transferred to the carbonyl oxygen with an O…H distance of 1.40 Å (other distances are as expected).

**Figure 11:**
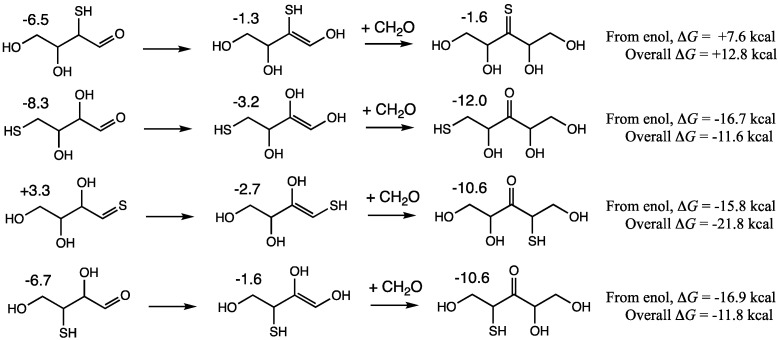
Thermodynamics of C_4_ + C_1_ → C_5_ aldol additions; *G*_rel_ of CH_2_O is +7.9 kcal.

Globally, the *G*_rel_ value for this retroaldol transition state of +22.2 kcal is on par with those of the C_2_ + C_1_ → C_3_ and C_3_ + C_1_ → C_4_ aldol addition reactions which range from +20.4 to +25.0. We therefore expect that the retroaldol could potentially compete kinetically with further aldol additions such as C_4_ + C_1_ → C_5_. If the C_4_ enol is **4e2**, addition of CH_2_O will lead to the thermodynamically less stable thione-pentose as shown in the first row of Figure 11. Thus, we expect the C_4_ → 2 C_2_ retro-aldol from **4a2** to be favored over further aldol addition of CH_2_O. This is a unique situation, because it is not true thermodynamically for other C_4_ + C_1_ → C_5_ additions where formation of the linear 3-ketopentoses (via **4e1**, **4e1-1** or **4e3-1**) is significantly exergonic as shown in Figure 11. The 3-ketopentoses however are dead ends in the formose cycle; they are thermodynamic sinks that do not undergo retroaldol C_5_ → C_3_ + C_2_ reactions in addition to removing C_4_ species from the pool—such reactions are parasites of the autocatalytic cycle. And as we have seen in Figure 7, since aldol additions to branched products are much less favorable, enolization of the 3-ketopentoses and addition of CH_2_O in a C_5_ + C_1_ → C_6_ reaction is much less likely.

### 3.6. When C_1_ Food Is Depleted

The scenarios discussed in the previous sections assume that the C_1_ food species is abundant. But what happens when it begins to deplete? Depending on the relative concentration of the various C_2_, C_3_, and C_4_ species, the following reactions could begin to be important: C_2_ + C_2_ → C_4_ aldoses (the opposite of the retroaldol), C_2_ + C_3_ → C_5_ aldoses or ketoses (depending on whether the C_3_ or C_2_ enolizes), C_2_ + C_4_ → C_6_ aldoses or ketoses (depending on whether the C_4_ or C_2_ enolizes), and C_3_ + C_3_ → C_6_ ketoses. This also opens up the possibility of incorporating more than one thiol group into a C_4_, C_5_ or C_6_ species.

Of the C_2_ + C_2_ → C_4_ reactions, the most favorable reaction between glycolaldehyde and mercaptoaldehyde is to form the 4-thioaldose **4a4** (see Figure 7). The reaction is overall exergonic (Δ*G* = −1.6 kcal) but the barrier is higher (Δ*G*^‡^ = +28.3 kcal overall, or +20.7 kcal from the enol) compared to aldol addition involving C_1_ food species which have barriers 2–5 kcal lower. If two mercaptoaldehyde molecules dimerized, this forms the 2,4-dithioaldoses. For this reaction, Δ*G* = +0.6 kcal overall to form 2,4-dithioerythrose (the threose is 0.1 kcal/mol less stable) and the overall barrier is 27.6 kcal/mol. Neither of these sulfur analogs is as thermodynamically favorable as the dimerization of glycolaldehyde (Δ*G* = −2.2 kcal) to form erythrose.

**Figure 12:**
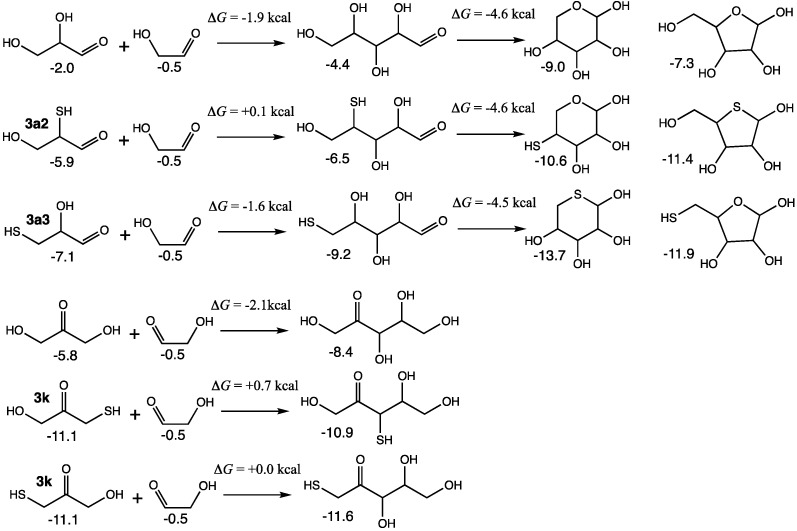
Thermodynamics of C_2_+ C_3_ → C_5_ aldol additions.

For the C_2_ + C_3_ → C_5_ reactions, we see a similar story. The non-sulfur analog reactions are thermodynamically more favorable in the forward direction (and therefore, less likely to undergo the corresponding retroaldol). In Figure 12, the addition of glycolaldehyde (via its enol) to glyceraldehyde to form open-chain ribose has Δ*G* = −1.9 kcal, which can favorably undergo ring-closure (the pyranose is more stable than the furanose; values shown are for the β anomers). Similarly, the addition of glycolaldehyde to dihydroxyacetone (via its enol) to form ribulose has similar thermodynamics with Δ*G* = −2.1 kcal (xylulose was less than 0.1 kcal different in free energy than ribulose).

For the sulfur analogs, the addition of **3a2** and glycolaldehyde to form the open-chain 4-thioribose has Δ*G* = +0.1 kcal. Ring closure to both the pyranose and furanose is favorable; having sulfur in the ring stabilizes the furanose in this case. Starting from **3a3** and forming 5-thioribose has Δ*G* = −1.6 kcal because thiols on the terminal carbon are favored. Ring closure is favorable and the pyranose is more stable than the furanose (see our previous work [19] for a more detailed discussion on the position of thiol groups in open chain aldoses and rings). Based on our discussion of Figure 7, we expect **3a3** to be less accessible because **3k** is the thermodynamic sink in that pathway. Thus, the more relevant sulfur analog is **3a2**, certainly less favorable than its non-sulfur counterpart. If the C_2_ species is mercaptoaldehyde, the results are similar but slightly less favorable.

For sulfur analogs forming C_5_ ketoses, **3k** can form two distinct enols and thus adding glycolaldehyde leads to 3-thioribulose (Δ*G* = +0.7 kcal) or 1-thioribulose (Δ*G* = 0.0 kcal). Both these reactions are thermodynamically less favorable than the non-sulfur counterpart (The corresponding thio-xyluloses are within 0.4 kcal of the thio-ribuloses. Also, having mercaptoaldehyde has similar but slightly less favorable thermodynamics as shown in Supplementary Materials). Another possibility is to add glycolaldehyde to the enol of **3a2** but the ketose product is a thione and the reaction is significantly endergonic.

**Figure 13:**
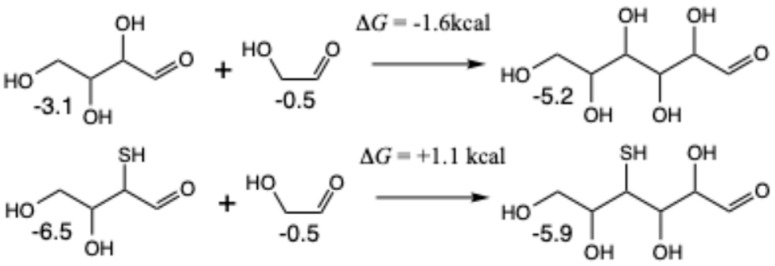
Thermodynamics of C_2_ + C_4_ (erythrose) → C_6_ (glucose) aldol additions.

For C_2_ + C_4_ → C_6_, as shown in Figure 13, we see the same trend. The aldol addition of erythrose and glycolaldehyde to form glucose is exergonic (Δ*G* = −1.6 kcal) while its sulfur analog is endergonic (Δ*G* = +1.1 kcal) starting from **4a2** (the most of the promising C_4_ aldehydes) and glycolaldehyde. If mercaptoaldehyde is used as the C_2_ species to form 2,4-dithioglucose, the reaction is more endergonic. Similar results are obtained for the C_3_ + C_3_ → C_6_ reactions forming fructose (see Supplementary Materials).

While C_1_ food is plentiful, the most favorable cross-Cannizzaro reaction, both thermodynamically and kinetically, involves the reduction of CH_2_O to CH_3_OH as discussed in the sections describing C_1_ and C_2_ reactions. When the food runs out, larger aldehydes could undergo Cannizzaro reactions which parasitize the autocatalytic cycle. A preliminary analysis of our calculations suggests that C_3_ and C_4_ species show similar energetics to the C_2_ species shown in Figure 6. Hence, while Cannizzaro reactions are thermodynamically favorable, they have larger barriers and are kinetically less favorable than the aldol addition reactions.

## 4. Conclusions

If H_2_S is incorporated as a thiol group in the formose reaction, its most salient contribution is utilizing mercaptoaldehyde as the C_2_ linchpin species. Both its enolization barrier and the entry into the cycle via first aldol addition (C_2_ + C_1_ → C_3_) are kinetically more favorable in the sulfur analog with barriers lowered by ~3 kcal. While there is no kinetic selectivity in forming the C_3_ species, there is significant thermodynamic selectivity for the aldehyde **3a2** over **3t1**. This could shunt the cycle through the reactions on the right hand side of Figure 7. While the initial C_3_ + C_1_ → C_4_ product is **4t2**, it more favorably enolizes to **4e2** (over **4e3-2**); thus, favoring the formation of **4a2**, the only C_4_ aldehyde that has a thermodynamically favorable C_4_ → 2 C_2_ retroaldol reaction (the barrier is also ~3 kcal lower than the non-sulfur analog). Thus, the presence of sulfur could accelerate the core autocatalytic cycle of the formose reaction compared to its non-sulfur analog, and this pathway is the most significant positive result of our work.

However, the messiness does not go away. A wide diversity of C_3_ and C_4_ compounds are accessible as shown in Figure 7. Having the thiol group in different positions provides selectivity for some species over others, but also adds more compounds to the mix. Sulfur analogs do not slow down the competing Cannizzaro reaction since its most likely channel is via reduction of CH_2_O to methanol, and reduces the C_1_ food. As CH_2_O depletes, sulfur analogs less favorably undergo further aldol additions to form C_4_, C_5_, and C_6_ species compared to their non-sulfur counterparts; although as concentrations of C_2_ and C_3_ build up, the equilibrium will shift towards larger species. It is unclear if slowing down the formation of larger species is favorable for kick-starting a proto-metabolism.

A question we asked but did not sufficiently answer in our previous work [19] was whether thiol groups could provide additional selectivity, especially if more than one thiol was present, and if there was a possibility that thiol groups could have served as precursor tags to phosphates in extant sugar metabolism. Having collected more data in this study, our answer at present is no. Forming dithiolated sugars is unfavorable, and even for monothiolated sugars, the thermodynamic favorability of the ketoses now causes these thermodynamic sinks to retard formose autocatalytic pathways. We are now considering if bisulfite analogs could lead to more pronounced selectivity instead of thiols.

Not included in this work is the intramolecular disproportionation of a thiolated sugar to form thioacids, or more promisingly, the addition of an organothiol to an aldehyde which disproportionates into a thioester. We are currently pursuing this possibility and expect to continue the story of the role of sulfur analogs in potential proto-metabolic autocatalytic cycles in a future publication.

## Data Availability

The data presented in this study are available in Supplementary Materials.

## Supplementary Materials

The following supporting information can be downloaded at: https://www.mdpi.com/article/10.3390/life15010001/s1, Energy breakdown of molecules and transition states; additional discussion sections and results referred to in main text.
