# Supplementary material for: Sulfur Analogs of the Core Formose Cycle: A Free Energy Map
Source: Life (Basel). 2024 Dec 24;15(1):1. doi: 10.3390/life15010001 (PMC11766735; doi:10.3390/life15010001)
Supplement: Supplementary file 1 [file life-15-00001-s001.zip › life-3366078-supplementary.pdf]

Supplementary Materials for:

Sulfur Analogs of the Core Formose Cycle: A Free Eenergy Map

By Jeremy Kua*, Maria T. Peña, Samantha N. Cotter, John Leca
University of San Diego, 5998 Alcala Park, San Diego, CA 92110, U.S.A.
*jkua@sandiego.edu

Part 1: Energy Breakdown Raw Data

Table S1 contains the energy breakdown for calculated structures in this article. The computational methods section describes each of these categories: E_{elec} is the electronic energy in atomic units. All other values are in kcal/mol. E_{solv} is the solvation energy when the molecule is embedded in a dielectric. H_{corr} includes the zero-point-energy and standard enthalpy corrections to 298 K. The entropic correction ($-0.5TS_{corr}$) is half of the standard gas-phase entropy correction at 298 K. The last column (G_{298}) is the total free energy obtained by adding the other four columns (where the atomic units of E_{elec} are converted to kcal/mol by multiplying 627.5096. The transition states are labeled "TS".

Table S1. Energy breakdown of molecules and transition states

	E_{elec} (a.u.)	E_{solv}	H_{corr}	$-0.5TS_{corr}$	G_{298}	G_{rel}
**Baseline (reference) molecules**						
H_2O	-76.44744	-8.66	15.74	-6.73	-47971.15	
H_2	-1.17957	1.54	8.39	-4.64	-734.90	
CO_2	-188.64112	-1.22	9.59	-7.61	-118373.35	
H_2S	-399.42206	-1.29	11.76	-7.33	-250638.04	
**C_1 molecules**						
CH_2O	-114.53629	-2.75	19.01	-7.79	-71864.15	7.87
$CH_2(OH)_2$	-191.00777	-10.33	38.94	-9.28	-119839.88	3.28
CH_3OH	-115.75737	-5.50	34.74	-8.47	-72618.09	-11.17
HCOOH	-189.81947	-6.96	23.81	-8.84	-119105.53	2.74
$CH_2(OH)(SH)$	-513.97545	-7.11	35.57	-10.01	-322506.07	3.98
HCOSH	-512.77342	-2.97	20.30	-9.43	-321762.34	12.81
HCSOH	-512.77109	-4.29	22.58	-9.26	-321759.75	15.40
CH_3COOH	-229.14542	-11.19	41.38	-9.69	-143770.45	
glycolic acid	-304.38296	-13.77	46.13	-10.86	-190981.73	
**C_2 molecules**						
glycolaldehyde (GA)	-229.11010	-7.26	41.58	-10.08	-143744.54	-0.50
**2a**	-552.08534	-6.19	38.55	-10.67	-346417.16	-6.23
**2a**_hydrate	-628.5534	-10.76	57.88	-11.67	-394387.84	-5.77
**2a**_addedH_2S	-951.52151	-5.31	54.75	-12.26	-597051.70	-2.74
$HOCH_2C(O)SH$	-627.36278	-9.15	42.96	-11.39	-393653.74	-6.57
$HSCH_2C(O)SH$	-950.31864	-4.17	39.68	-11.95	-596310.51	3.55
**2e**	-229.09642	-9.21	41.54	-9.88	-143737.75	6.29
**2t**	-552.07183	-4.95	40.22	-10.55	-346405.65	5.28

**C_3 molecules**						
**3a2**	-666.65298	-6.90	61.41	-12.20	-418288.83	-5.91
**3e2**	-666.64130	-8.05	61.73	-12.43	-418282.55	0.37
**3t2**	-666.63548	-10.74	62.65	-12.52	-418280.77	2.18
**3t1**	-666.63650	-8.20	62.81	-12.23	-418278.42	4.53
**3e1-1**	-666.64367	-8.24	61.55	-12.37	-418284.36	-1.41
**3k**	-666.65469	-10.21	60.95	-12.58	-418294.06	-11.14
**3e1-2**	-666.64090	-10.46	61.34	-12.58	-418285.26	-2.31
**3a1**	-666.65318	-7.43	60.96	-12.35	-418290.09	-7.13
**C_4 molecules**						
**4ba2**	-781.21151	-10.78	83.63	-13.91	-490158.78	-3.81
**4t2**	-781.20031	-12.97	85.15	-13.96	-490152.47	2.50
**4e2**	-781.19856	-16.27	83.88	-14.22	-490156.20	-1.23
**4a2**	-781.21466	-11.64	83.79	-13.94	-490161.49	-6.52
**4r2**	-781.21018	-15.73	84.18	-13.52	-490161.96	-6.99
**4e-3-2**	-781.19757	-15.97	84.08	-14.18	-490155.04	-0.07
**4k3**	-781.22170	-10.88	83.71	-13.96	-490165.25	-10.27
**4e-3-1**	-781.19892	-16.23	83.72	-14.24	-490156.57	-1.60
**4a3**	-781.21468	-11.22	83.37	-14.11	-490161.67	-6.69
**4r3**	-781.21516	-13.27	84.33	-13.48	-490162.43	-7.46
**4ba3**	-781.21698	-10.96	83.25	-14.03	-490162.89	-7.92
**4k1**	-781.22295	-11.14	83.58	-14.09	-490166.55	-11.58
**4e4-2**	-781.20367	-14.49	83.86	-14.34	-490157.77	-2.80
**4k4**	-781.21890	-13.51	83.47	-14.23	-490166.63	-11.66
**4e4-1**	-781.21002	-11.35	83.88	-13.95	-490158.20	-3.23
**4a4**	-781.21499	-12.47	83.24	-14.09	-490163.23	-8.25
**4r4**	-781.21540	-18.62	85.63	-13.30	-490166.45	-11.48
**4e1**	-781.20637	-13.07	83.97	-14.10	-490157.70	-2.73
**4t1**	-781.20066	-11.92	85.07	-13.89	-490151.65	3.32
**4r1**	-781.20504	-13.76	84.22	-13.45	-490156.65	-1.68
**C_5 molecules**						
3-thionepentose	-895.77025	-16.86	107.58	-14.85	-562028.56	-1.57
1-thio-3-ketopentose	-895.78768	-14.07	106.04	-15.60	-562039.00	-12.01
2-thio-3-ketopentose	-895.78595	-14.1	106.37	-15.59	-562037.60	-10.61
ribose (linear)	-572.80465	-17.68	108.81	-15.24	-359364.52	-4.43
ribopyranose	-572.81217	-20.15	110.08	-13.92	-359369.13	-9.03
ribofuranose	-572.80602	-21.03	109.73	-14.82	-359367.40	-7.30
4-thioribose (linear)	-895.77868	-14.17	105.91	-15.55	-562033.53	-6.54
4-thioribopyranose	-895.7885	-14.16	106.99	-14.51	-562037.56	-10.57
4-thioribofuranose	-895.77381	-24.32	107.8	-15.19	-562038.37	-11.38
5-thioribose (linear)	-895.78433	-13.03	105.65	-15.50	-562036.15	-9.16
5-thioribopyranose	-895.79279	-16.24	108.39	-14.31	-562040.73	-13.74
5-thioribofuranose	-895.78012	-19.62	106.51	-15.12	-562038.85	-11.86
ribulose	-572.80921	-18.24	108.77	-15.78	-359368.52	-8.43
3-thioribulose	-895.78416	-15.11	106.07	-15.64	-562037.84	-10.85
1-thioribulose	-895.78608	-14.26	105.85	-15.77	-562038.54	-11.55

**C_6 molecules**						
glucose (linear)	-687.36989	-20.43	131.29	-17.00	-431237.34	-5.22
4-thioglucose (linear)	-1010.34144	-17.29	128.2	-16.83	-633904.87	-5.86
**Transition States**						
Figure 3 (row 1)	-305.51325	-9.50	55.01	-11.15	-191678.14	37.01
Figure 3 (row 2)	-628.47394	-7.34	51.40	-11.70	-394341.07	40.97
Figure 3 (row 3)	-628.46524	-12.01	52.37	-11.71	-394339.32	42.73
Figure 6 (row 1)	-743.05540	-9.81	73.94	-13.45	-466223.71	30.35
Figure 6 (row 2)	-1066.01656	-6.62	70.59	-14.13	-668885.79	35.17
**2a ←→ 2e**	-781.43720	-20.64	85.89	-14.95	-490309.04	15.28
**2e ←→ 2t**	-704.96182	-19.65	71.70	-13.42	-442331.67	21.52
**2e ←→ 3a2**	-819.52890	-14.44	90.82	-14.98	-514200.85	24.38
**2e ←→ 3t1**	-819.51903	-20.27	90.97	-15.19	-514200.55	24.68
**3a2 ←→ 3e2**	-896.00422	-19.04	109.76	-16.51	-562177.03	19.30
**3e2 ←→ 3t2**	-819.52564	-22.14	94.12	-15.11	-514203.33	21.86
**3t1 ←→ 3e1-1**	-895.99608	-26.04	111.63	-16.52	-562177.08	19.26
**3e1-1 ←→ 3k**	-896.00032	-21.90	107.85	-16.43	-562179.28	17.06
**3k ←→ 3e1-2**	-895.99978	-23.72	110.11	-17.62	-562179.69	16.65
**3e1-2 ←→ 3a1**	-895.99880	-18.99	107.43	-16.30	-562175.71	20.63
**3e2 ←→ 4t2**	-934.08423	-25.08	114.15	-17.12	-586074.87	22.38
**3e1-1 ←→ 4k3**	-934.08518	-20.86	111.28	-17.16	-586074.17	23.08
**3e1-1 ←→ 4ba3**	-934.07731	-24.17	111.52	-17.15	-586072.28	24.97
**3e1-2 ←→ 4ba3**	-934.09054	-21.40	113.12	-16.61	-586075.66	21.59
**3e1-2 ←→ 4k1**	-934.09796	-17.49	114.03	-16.30	-586075.20	22.05
**4t2 ←→ 4e2**	-1010.56292	-22.05	129.65	-17.88	-634048.21	20.17
**4e2 ←→ 4a2**	-1010.56633	-20.49	129.10	-17.94	-634049.40	18.98
**4a2 ←→ 2a + GA**	-934.08737	-21.23	112.24	-17.27	-586075.05	22.19
**4t2 ←→ 4e3-2**	-1010.555509	-29.10	133.94	-17.67	-634046.11	22.28
**4e3-2 ←→ 4k3**	-1010.552243	-30.14	129.62	-18.53	-634050.28	18.11
**4k3 ←→ 4e3-1**	-1010.56132	-21.61	131.89	-17.97	-634044.62	23.76
**4e3-1 ←→ 4a3**	-1010.56343	-21.25	131.29	-18.01	-634046.23	22.16
**4k1 ←→ 4e4-2**	-1010.54961	-32.07	129.45	-17.83	-634050.02	18.36
**4e4-2 ←→ 4k4**	-1010.56360	-22.84	132.13	-18.14	-634047.21	21.17
**4k4 ←→ 4e4-1**	-1010.56186	-20.87	130.21	-17.91	-634045.84	22.54
**4e4-1 ←→ 4a4**	-1010.56022	-19.08	127.42	-17.17	-634045.07	23.32
**4a4 ←→ 2a + GA**	-934.09628	-16.24	112.07	-17.11	-586075.67	21.58
**4k1 ←→ 4e1**	-1010.56633	-20.49	129.10	-17.94	-634049.40	18.98
**4e1 ←→ 4t1**	-1010.56345	-21.45	132.88	-17.93	-634044.76	23.62
**4t1 ←→ 2a + GA**	-934.08468	-29.32	114.83	-17.36	-586078.95	18.30

Part 2: A Note about Solvation

In a few rare cases, the solvation energy calculation either failed or looked spurious compared to similar transition states. In these cases, we made an empirical correction. Only one calculation failed (**4t1 <-> 2a + GA**) and we're not sure why, but we think there was a cusp or numerical instability in generating the molecular surface. By changing some parameters in the calculation, we were able to complete the calculation. By doing the same with the five other analogous reactions (which were successful using out normal protocol), we made a solvation correction for this one case. In a couple of cases involving C_4 thione-to-enol isomerization, the solvation energies looked spurious and very different from the other enolization transition states. We recalculated the solvation energy using the SMD model and applied empirical corrections for consistency.

Part 3: Cis and Trans Enolization Energy Comparisons

In all cases, the cis-enol allowing for an intramolecular hydrogen bond is thermodynamically slightly favored over the trans-enol by 1-2 kcal. For the transition states, the cis pathway is kinetically favored in all cases by 2-4 kcal except one – the conversion of **3e1-1** to **3k** where the trans has a transition state energy 0.8 kcal lower. The energies shown in Figure S1 are the G_{rel} values in kcal.

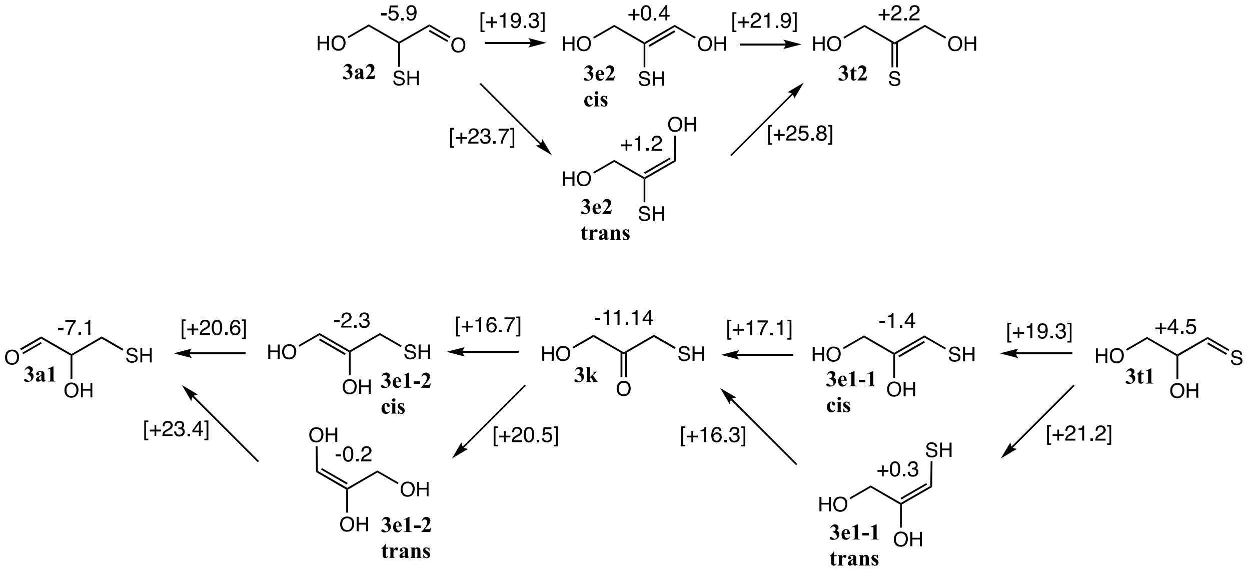

Figure S1. Relative Free Energies for cis versus trans C_3 enolizations (G_{rel} values in kcal)

Part 4: D-erythrose versus D-threose Energy Comparisons

In the open chain aldehydes, threose is marginally more stable with the thiol in the 2,3,4 positions. For the aldehyde-thione, threose is marginally less stable. The difference in relative free energy in three of these cases is less than 0.5 kcal (within the computational error) and only in **4a4** is threose clearly more stable than erythrose. The β-furanoses are all more stable than the open chain aldehyde; threose is marginally more stable than erythrose in three of the four cases (**4r4** being the exception) but the free energy differences are small. For the retroaldol $C_4 \rightarrow 2\ C_2$ reaction, threose is kinetically less favorable than erythrose in all cases. As discussed in the main paper, we were not able to cleanly locate a transition state for **4a3 → 2t + GA**. The energies are shown in Figure S2 and G_{rel} values are in kcal.

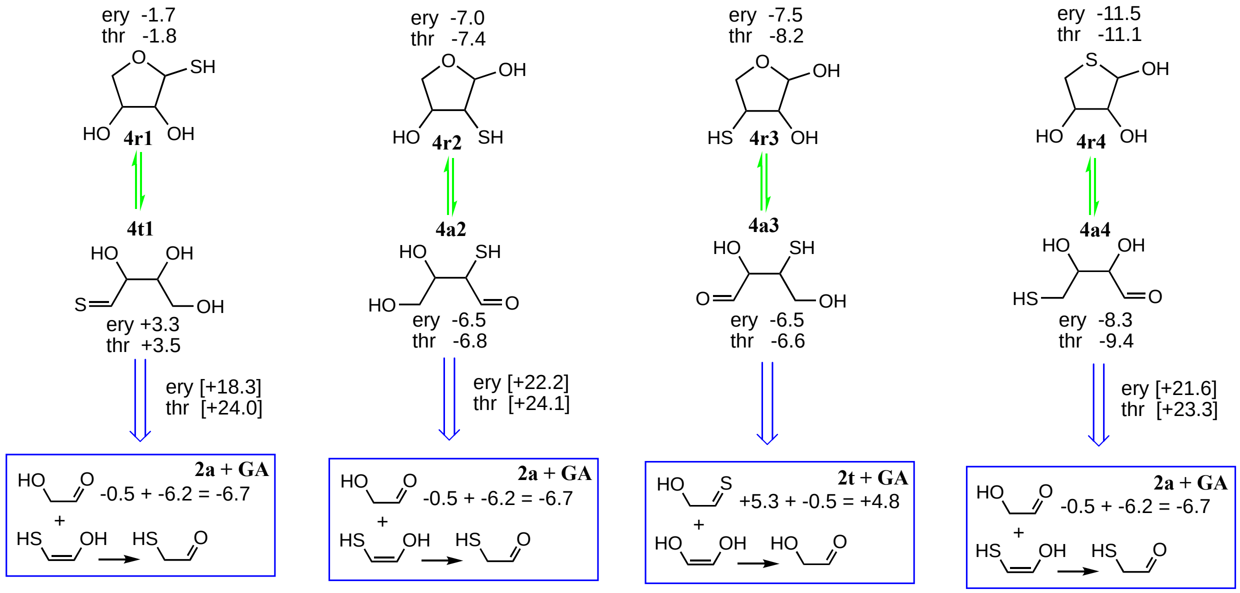

Figure S2. Relative Free Energies for D-erythrose versus D-threose (G_{rel} values in kcal)

Part 5: Retroaldol of **4a3** forms 4-center ring intermediate

We were not successful in cleanly optimizing a transition state for the retroaldol reaction of **4a3 → 2t + GA**. In Figure S3 on the left is a reasonable guess transition state, and on the right is the intermediate structure that results. This structure that contains a four-membered ring has a G_{rel} of +0.6 kcal. Attempts to find transition states between the intermediate and the reactant or product resulted in falling to one of the three minima (reactant, product, intermediate). Since this retroaldol reaction is overall endergonic by +11 kcal, we abandoned the effort as this reaction is unlikely to take place in the overall network.

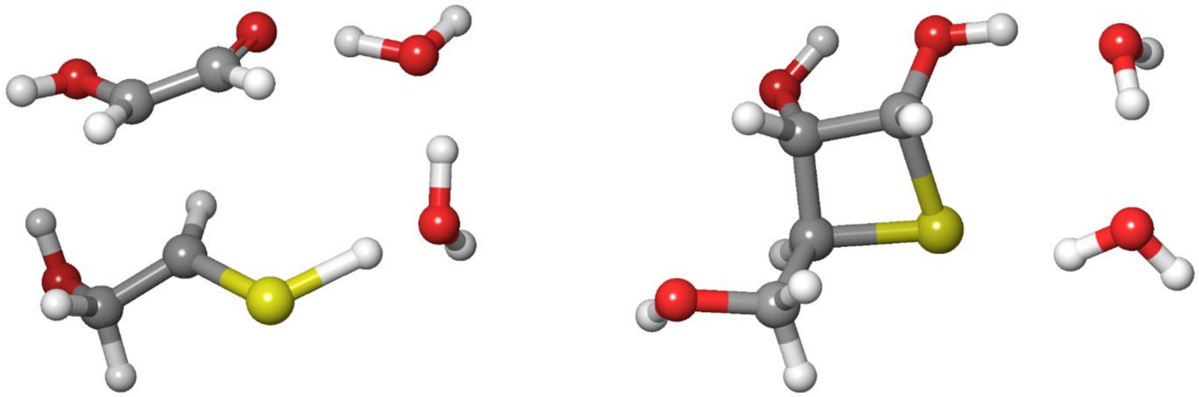

Figure S3. Retroaldol transition state attempts form a ring intermediate

Part 6: $C_3 + C_2 \rightarrow C_5$ Aldol Additions: Other isomers

Thermodynamics of the formation of xyluloses is less than 0.5 kcal different from forming the ribuloses (Figure 12 in the main text) in all cases as shown in Figure S4.

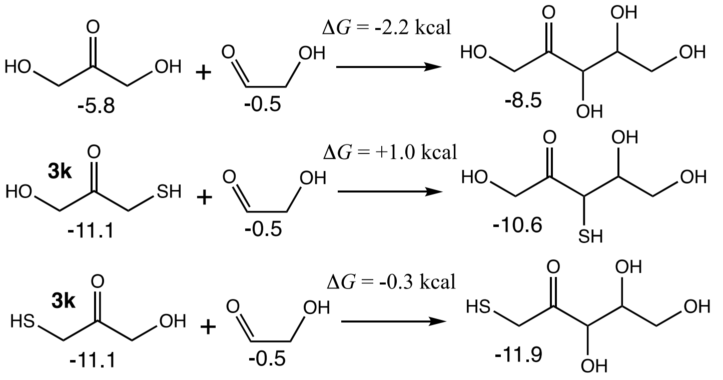

Figure S4. Formation of D-xylulose from $C_3 + C_2$ aldol addition (G_{rel} values in kcal)

The formation of D-arabinose, D-xylose, and D-lyxose is thermodynamically similar to ribose. While there is a marginal preference for ribose in the sulfur analogs in several cases, the differences in G_{rel} are small. In some cases, arabinose or xylose is the most stable of the four but there is no clear trend comparing the open chain to the β-pyranoses or β-furanoses, as shown in Figure S5.

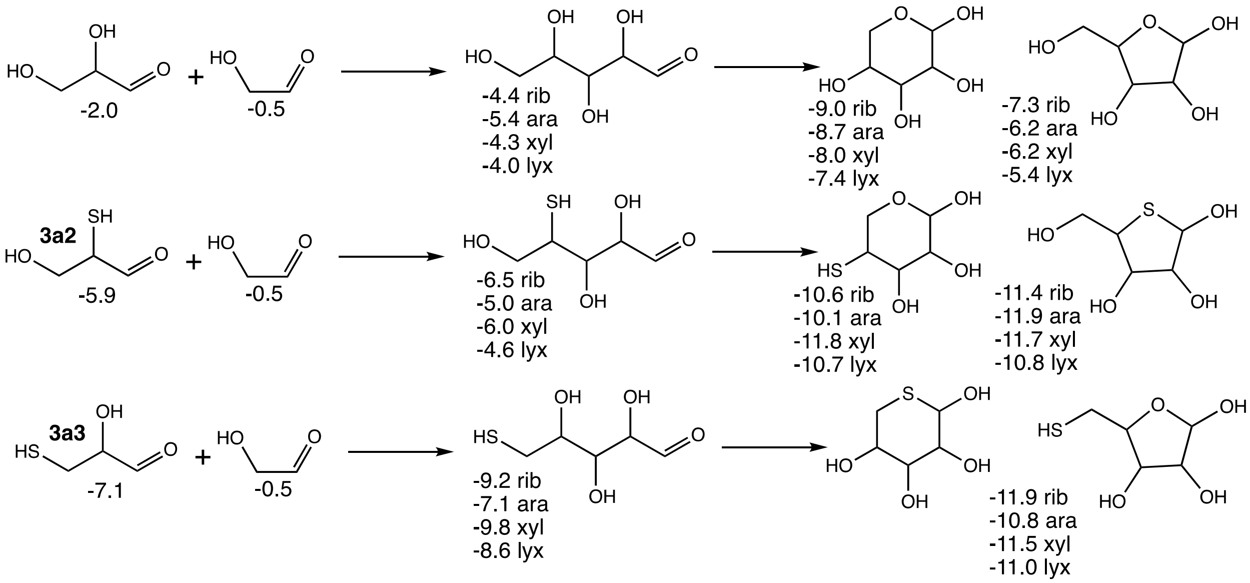

Figure S5. Formation of D-aldoses from $C_3 + C_2$ aldol addition (G_{rel} values in kcal)

Part 7: $C_3 + C_3 \rightarrow C_6$ Aldol Additions to D-Fructose

While the $C_3 + C_3 \rightarrow C_6$ aldol addition to form linear D-fructose is thermodynamically favorable starting from dihydroxyacetone and D-glyceraldehyde (first row of Figure S6), sulfur analogs result in a slightly endergonic reaction. Subsequent ring closure to the pyranose is exergonic, but in most cases is not sufficient to make the overall reaction exergonic. An exception is the formation of 1,3-dithiofructose (last row of Figure S6) where the overall reaction is mildly exergonic with ΔG = +2.1 + (-17.4+14.9) = -0.4 kcal. We did not include 3-thioglyceraldehyde (**3a3**) as a reactant because it is less likely to be formed compared to 2-thioglyceraldehyde (**3a2**); see Figure 7 and the main text for discussion.

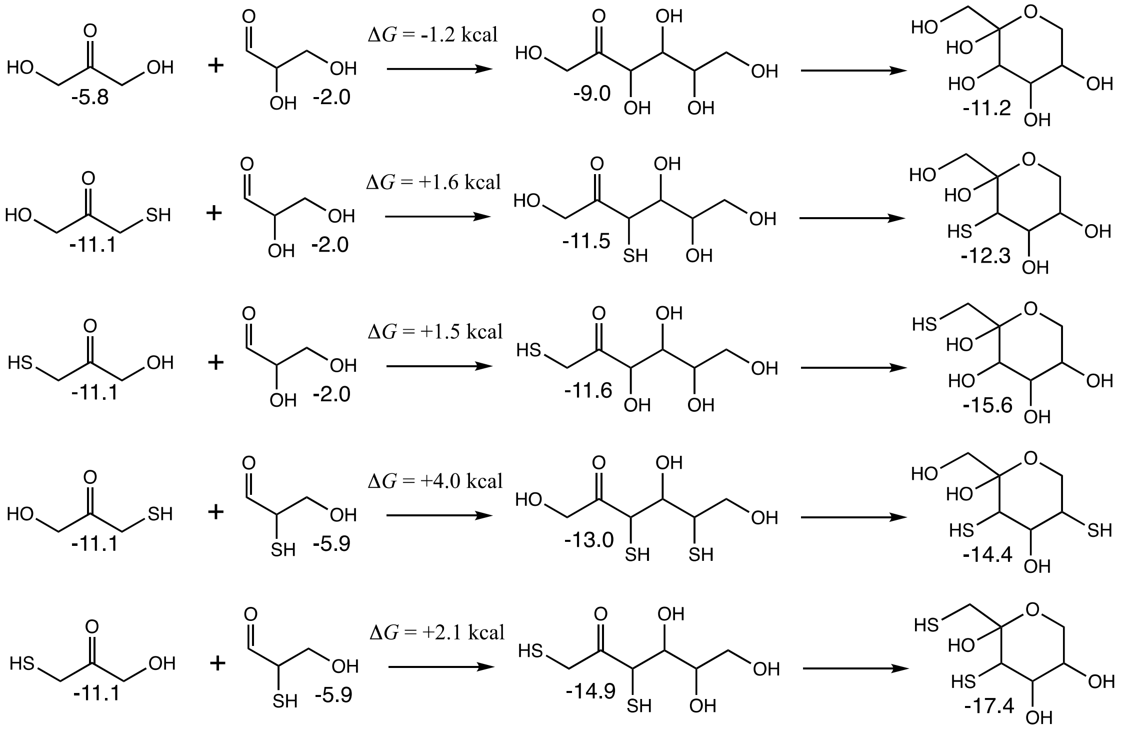

Figure S6. Formation of D-fructoses from $C_3 + C_3$ aldol addition (G_{rel} values in kcal)
